# Choline – a scoping review for Nordic Nutrition Recommendations 2023

**DOI:** 10.29219/fnr.v67.10359

**Published:** 2023-12-21

**Authors:** Rima Obeid, Therese Karlsson

**Affiliations:** 1 Department of Clinical Chemistry and Laboratory Medicine, University Hospital of the Saarland, Homburg, Germany; 2 Department of Internal Medicine and Clinical Nutrition, Sahlgrenska Academy, University of Gothenburg, Gothenburg, Sweden

**Keywords:** phosphatidylcholine, intake, brain, liver, cardiovascular disease

## Abstract

Choline is an essential nutrient with metabolic roles as a methyl donor in one carbon metabolism and as a precursor for membrane phospholipids and the neurotransmitter acetylcholine. Choline content is particularly high in liver, eggs, and wheat germ, although it is present in a variety of foods. The main dietary sources of choline in the Nordic and Baltic countries are meat, dairy, eggs, and grain. A diet that is devoid of choline causes liver and muscle dysfunction within 3 weeks. Choline requirements are higher during pregnancy and lactation than in non-pregnant women. Although no randomized controlled trials are available, observational studies in human, supported by coherence from interventional studies with neurodevelopmental outcomes and experimental studies in animals, strongly suggest that sufficient intake of choline during pregnancy is necessary for normal brain development and function in the child. Observational studies suggested that adequate intake of choline could have positive effects on cognitive function in older people. However, prospective data are lacking, and no intervention studies are available in the elderly.

## Popular scientific summary

- Choline is an essential nutrient involved in many biochemical reactions in the body and is a precursor for phospholipids in the cell membrane.
- Meat, dairy, eggs, and grains are the main dietary sources in Nordic and Baltic diets.
- Deficiency of choline can cause liver and muscle dysfunctions.
- There is suggestive evidence for a role of maternal choline intake in normal child brain development and function.
- A qualified biomarker for assessing choline status has not been established.

Choline is a water-soluble quaternary amine with a molecular weight of 104.2 g/mol. Foods contain water-soluble (free choline, phosphocholine, and glycerophosphocholine) and lipid soluble choline compounds (phosphatidylcholine and sphingomyelin). Phosphatidylcholine accounts for approximately 95% of total choline in animal tissues. The remaining 5% of tissue choline consists of free choline, phosphocholine, glycerophosphocholine, Cytidine 5’-diphosphocholine (CDP-choline), and acetylcholine (1). Choline is oxidized to betaine. Betaine supports folate as an alternative methyl donor in one carbon metabolism that plays a central role in cell metabolism and DNA-methylation. In addition, choline is needed to produce phospholipids that are major constituent of cell membranes and play a role in hepatic lipid metabolism. Choline is also used to produce the neurotransmitter acetylcholine. Therefore, choline is related to major metabolic pathways (one-carbon and lipid) that have been associated with chronic diseases. Choline has been recognized an essential nutrient in human by the Food and Nutrition Board of the US National Academy of Sciences of the US Institute of Medicine (2) and later on confirmed by European Food Safety Authority Panel on Dietetic Products, Nutrition and Allergies (3). A small amount of choline can be synthesized de-novo from phosphatidylethanolamine via phosphatidylethanolamine methyl transferase (PEMT) in the liver. However, depletion-repletion studies have provided evidence that dietary restriction of choline in humans causes liver and muscle damage, while feeding choline can avert these symptoms (4). Therefore, the endogenous synthesis of choline is not sufficient, and dietary sources of choline are necessary to maintain health, making choline an essential nutrient for humans.

The adequate intake (AI) levels for choline for different age groups and life stages have been defined by the IOM (present National Academies of Sciences, Engineering, and Medicine) in 1998 (Table 1). In 2016, the EFSA Panel considered that average requirements and population reference intakes for choline could not be derived for adults, infants, and children, and instead AIs (3) were defined (Table 1). The aim of this scoping review is to describe the present evidence on a potential role for choline in health-related outcomes upon which dietary reference values (DRVs) in the Nordic Nutrition Recommendations 2023 could be based (Box 1).

**Table 1:** Adequate intake levels choline as set by EFSA and IOM panels

Life stage	IOM – 1998			EFSA – 2016	
	Age	AI (mg/d)		Age	AI (mg/d)
		Males	Females		
Infants	0–6 months	125	125	0–6 months	120
	7–12 months	150	150	7–11 months	160
Children	1–3 years	200	200	1–3 years	140
	4–8 years	250	250	4–6 years	170
	9–13 years	375	375	7–10 years	250
	14–18 years	550	400	11–14 years	340
				15–17 years	400
Adults	≥19 years	550	425	≥18 years	400
Pregnancy	–	–	450	–	480
Lactation	–	–	550	–	520
LOAEL	–	7,500	7,500	7,500	7,500
UL	–	3,500	3,500	3,500	3,500

AI: adequate intake; EFSA: European Food Safety Authority; IOM: Institute of Medicine; LOAEL: Lowest Observed Adverse Effect Level; UL: Tolerable Upper Intake Level.

Box 1 Background papers in Nordic Nutrition Recommendations 2023

- This paper is one of many scoping reviews commissioned as part of the Nordic Nutrition Recommendations 2023 (NNR2023) project (6)
- The papers are included in the extended NNR2023 report, but, for transparency, these scoping reviews are also published in Food & Nutrition Research
- The scoping reviews have been peer reviewed by independent experts in the research field according to the standard procedures of the journal
- The scoping reviews have also been subjected to public consultations (see report to be published by the NNR2023 project)
- The NNR2023 committee has served as the editorial board
- While these papers are a main fundament, the NNR2023 committee has the sole responsibility for setting dietary reference values in the NNR2023 project

## Methods

Based on initial literature searches (5) and public consultations conducted end of 2020, the NNR2023 Committee decided to incorporate a background review on choline for the first time. Choline was not selected for a *de novo* systematic review. In the literature search of the NNR2023 Committee, the EFSA Panel Publication from 2016 (3) was identified as a reliable source of evidence, since it was based on systematic literature review. The present scoping review on choline is based on the evidence as judged by the EFSA Panel. In addition, we reviewed the literature cited there and the original report of the IOM where choline was first considered an essential nutrient. We searched in PubMed to identify articles published after the EFSA report (in 2016), addressing the association between choline intake and health outcomes relevant to Nordic countries. During preparation of this review (March to September 2021), we searched PubMed using terms containing ‘choline’[MeSH Terms] OR ‘choline’[All Fields]) in combination with several MeSH terms that cover the outcomes of interest, such as ‘cardiovascular diseases’, ‘stroke’, ‘pregnancy’, ‘cognitive function’, ‘memory’, ‘dementia’, ‘pregnancy’, ‘lactation’, ‘birth defects’, ‘brain development’, or ‘liver function’. The authors conducted the search in different health areas with a time limit between 2016 and 2021. The most relevant articles identified by this non-systematic review were included in this review. The EFSA report was assessed using a modified AMSTAR 2 tool (6, 7) and based on information from the EFSA report 2016 (3) and an external scientific report describing literature search and review process (8). Based on the NNR-modified AMSTAR 2 and the procedure for overall rating proposed by the NNR2023 Committee, the EFSA review was judged to be of critically low confidence due to flaws in relation to lack of information of predetermined methods, selection/data extraction in duplicate, and sources of funding. However, the report is based on a comprehensive literature search, appropriate risk of bias assessment, and justification of excluded studies. Therefore, we consider it to be a reliable source of evidence.

## Physiology and metabolism

### Choline absorption and tissue distribution

Choline in the diet is actively taken up by the enterocytes via the saturable organic cation transporters choline transporter-like protein 1 (CTL1) also called solute carrier family 44 member 1 (SLC44A1). After ingesting phosphatidylcholine (main choline storage form in animal tissues), plasma-free choline raises to show a peak level after 3–4 h, and it is cleared within approximately 8 h (9). The hepatic ABCB4 is responsible for excretion of choline-containing phospholipids such as phosphatidylcholine into the bile. Bile phosphatidylcholine constitutes an important source of body choline needs (10). Lyso-phosphatidylcholine that is produced from phosphatidylcholine hydrolysis can be partly recycled through incorporation into chylomicrons that is later absorbed in the intestinal epithelial cells. Phospholipases convert dietary and bile phosphatidylcholine to choline. Water-soluble choline forms may enter the portal circulation unchanged. The recycled amount of phospholipids is regulated by the amount of fat in the diet. Recent studies in mice have shown that endogenous synthesis of phosphatidylcholine in the enterocytes is enhanced in response to high fat diet feeding, while bile phosphatidylcholine is not sufficient under these circumstances (11). The unabsorbed choline is converted by gut bacteria to trimethylamine (TMA) that enters the blood stream and is metabolized in the liver to trimethylamine-N-oxide (TMAO). Recent studies have shown that free choline (as bitartrate or chloride salts), but not dietary phosphatidylcholine, caused a temporary raise of plasma TMAO in human (12) and animal studies (13). Therefore, the proportion of choline absorbed from the diet or supplements may depend on the form of choline in the diet, bile secretion, and the amount of fat in the diet. More studies in human are needed to estimate the contribution of the liver and enterocytes endogenous phosphatidylcholine synthesis, recycling of bile choline and degradation of choline by gut bacteria in body choline requirements.

Several types of cellular transporters are responsible for universal or tissue-specific distribution of free choline. CTL1, or SLC44A1, is a low-affinity universal choline transporter present in all tissues, such as kidney and placental tissues, enterocytes, hepatocytes, mitochondria, and synaptosomes. This transporter provides the cell with choline needed for the synthesis of phospholipids and betaine. Presynaptic cholinergic nerve terminals are rich in a high-affinity choline transporter (CHT; solute carrier family 5 member 7 encoded by SLC5A7), which is a carrier-mediated sodium-, chloride-, and ATP-dependent saturable uptake system. The third choline transporter (OCT1-3: a member of the solute carrier 22 family SLC22A1-3) is present in the blood–brain barrier and erythrocyte membranes, and it has a high affinity for choline (14). The mfsd2s transporter in endothelium of the blood–brain barrier of micro-vessels has been shown to take up plasma lyso-phosphatidylcholine carrying long-chain fatty acids. This transport path contributes significantly to brain phospholipids (15).

Saturable and non-saturable choline uptake mechanisms are operating in the mammary epithelium, with the non-saturable system operating at higher maternal choline supply. The mammary epithelium is capable of converting free choline to other choline-containing compounds. Choline is transported from the mother to the fetus across the placenta (16) via a specific transport system on both the maternal and fetal sides of the syncytiotrophoblast (17).

### Choline metabolism

**Fig. 1:**
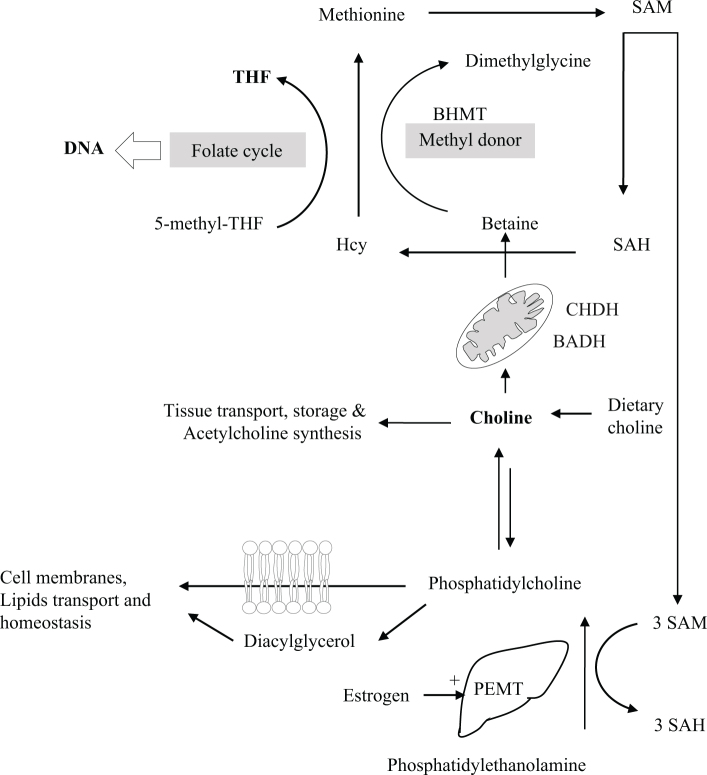
Choline metabolism. BADH: betaine aldehyde dehydrogenase; BHMT: betaine-homocysteine methyltransferase; CHDH: choline dehydrogenase; Hcy: homocysteine; PEMT: phosphatidylethanolamine N-methyltransferase; SAH: S-adenosylhomocysteine; SAM: S-adenosylmethionine; THF: tetrahydrofolate (Obeid R, unpublished figure).

Choline is transported into the mitochondria where it is oxidized to betaine in a two-step enzymatic reaction mediated by the mitochondrial choline dehydrogenase (CHDH) and betaine aldehyde dehydrogenase (BADH) (mitochondrial or cytoplasmic) (Fig. 1). This reaction occurs mainly in the liver and kidney. Betaine is a methyl donor in one-carbon metabolism, and thus, it interacts with other nutrients such as folate, vitamin B_12_, riboflavin, and the amino acid methionine. Metabolisms of choline and folate show interdependency (18–20). Both folate and choline (via betaine) are methyl donors and cooperative negative determinants of plasma total homocysteine (tHcy) (21–24).

Besides its role in one carbon metabolism, choline is required for synthesizing phospholipids such as phosphatidylcholine, which is the main storage form of choline and an essential component of cell membranes. Phosphatidylcholine is needed for lipoprotein assembly and secretion that are required for normal hepatic secretion of very low-density lipoprotein (VLDL) from the liver, thus explaining that choline deficiency is associated with fatty liver. Moreover, choline is used to produce the neurotransmitter acetylcholine (mainly in the brain, heart, kidney, and placenta).

The gene encoding PEMT enzyme is upregulated by estrogen that binds and activates estrogen-responsive elements in the gene (25). In females, the endogenous production of choline is subject to hormonal upregulation, which was the argument used by IOM to define higher AIs for males compared to females. Moreover, in addition to polymorphisms in genes involved in one-carbon metabolism, several polymorphisms in the PEMT gene have been described to have a potential effect on individuals’ predisposition to choline deficiency (26). However, the so far available studies on eventually higher requirement of subjects with certain genotypes did not provide sufficient evidence to justify the need to set higher intake recommendations of choline for the whole population.

## Assessment of nutrient status

There are currently no optimal blood biomarkers that can accurately mirror choline deficiency or sufficiency. A biomarker should optimally show a dose-dependent association with the intake of choline. Several biomarkers have been measured in plasma/serum or breast milk in clinical studies. Examples of these markers are free choline (27), total choline (including choline esters) (28), and other choline containing compounds, such as phosphatidylcholine, glycerophosphocholine, sphingomyelin, and phosphocholine (29, 30). The last four derivatives are highly available in breast milk and have been used in previous studies to compute total choline by summing up the single components (30, 31).

Higher choline intake from foods and/or supplements increases plasma free choline and betaine in adults (32, 33) and in pregnant females (i.e. 16 week of gestation) (34), suggesting that the concentrations of these biomarkers could be a surrogate measure of maternal choline supply. Plasma concentrations of free choline decline in people fed a diet that is deprived of choline (4). The levels raise after feeding a meal that is rich in choline (i.e. three eggs) (32) or after providing choline in supplemental forms (i.e. phosphatidylcholine or choline bitartrate) (32, 35, 36). When choline is absorbed in the intestine, it is distributed to tissues, stored, engaged in lipid transport, or eliminated via the kidney within approximately 8 h. Although fasting plasma concentrations of free choline and betaine show response to choline depletion (within 1–3 weeks) and repletion (acute and chronic), they may not accurately reflect small variations in dietary intakes and are thus not suited as markers of choline intake in clinical studies (37). The use of fasting plasma choline as an exposure variable in clinical studies may underestimate between-individual variations in choline intake. Summing up several forms of concentrations of choline derivatives (i.e. free choline, phosphatidylcholine, phosphocholine, etc.) is more likely to increase the accuracy of estimating choline content in breastmilk, for example. There is certainly a need to search for surrogate markers or a combination of markers that reflect choline status.

## Dietary intake in Nordic and Baltic countries

Choline content is particularly high in liver, eggs, and wheat germ although it is present in a variety of other foods. Also, the food additive lecithin, which is rich in phosphatidylcholine, can contribute to dietary choline. The main dietary sources of choline in the Nordic and Baltic countries are meat, dairy, eggs, and grain (38). In food, choline is present either as free choline or in the esterified forms of phosphatidylcholine, phosphocholine, glycerophosphocholine, and sphingomyelin (39). Generally, a plant-based diet contains less choline than an animal-based diet (40). The global trend to reduce animal-source foods in order to attain sustainability goals implies that it may be difficult to achieve AIs of choline, especially in vulnerable population groups, such as young women and infants. A ‘vegetarian tendency’ dietary pattern was associated with lower intake coefficients for choline in women of childbearing age (41). There is no sufficient evidence of intakes of total methyl-group donors (methionine, choline, betaine, and folate) and co-factors (riboflavin and vitamins B_6_ and B_12_) in relation to choline sufficiency in vegans and vegetarians. Sources of foods need to be considered in achieving AIs for choline moving toward a more plant-based diet.

Data on choline intake in the Nordic and Baltic countries are scarce, and results from national population surveys are available only from one publication (38). In Swedish and Finnish adults aged 18 to ≥75 years, average reported choline intake ranged from 317 to 468 mg/day in men and 317 to 404 mg/day in women (38). In children, average choline intake ranged from 171 to 180 mg/day in Finnish children aged 1 to <3 years, 256–285 mg/day in children aged 3 to < 10 years, and 292–373 mg/day in children between 10 and <18 years (38).

Compared to other populations in Europe, the average reported intake of choline seems to be slightly higher in Nordic countries (38). However, reported mean choline intakes in Nordic countries were lower than the AIs, especially in vulnerable groups such as young women and pregnant and lactating women. This implies that a large proportion of females in fertile age are not achieving optimal daily choline intake. For example, a national survey in Latvia has shown that estimated median intake of total choline was 356 [5th, 95th percentiles = 200, 592] mg/day in pregnant women and 288 mg/day in pregnant adolescents (38). These average intake values are similar to those among women from Sweden (*n* = 807) [median (5th, 95th percentile) = 356 (186, 631) mg/d] and women from Finland (*n* = 710) [327 (177, 587) mg/d], implying that choline may be under consumed on a population level. However, the only existing food database with choline as of today is the U.S. Department of Agriculture (USDA) database (39). There are no food composition data available in any of the Nordic or Baltic countries, and national databases of choline content in foods are warranted. Thus, there is some uncertainty in the estimated choline intake from Nordic (and European) populations. There is also uncertainty about the health consequences of not achieving the AI level of choline such as during pregnancy.

## Health outcomes relevant for Nordic and Baltic countries

### Pregnancy and lactation and infant’s health

To calculate the additional need for dietary choline during pregnancy, the IOM estimated choline transfer from the mother to the fetus and of choline accretion in the fetus and placenta during pregnancy, and this estimate was added to the requirements for non-pregnant females to get an AI of 450 mg/d for pregnant women. The EFSA panel considered that the approach followed by IOM is not feasible to set DRVs for pregnant females due to a lack of data. The EFSA panel recognized that choline requirement in pregnancy is higher than in non-pregnant females, and that increased loss of choline in urine occurs during pregnancy. The AI for pregnant females (480 mg/d) was based on isometric scaling from the AI of non-pregnant females (400 mg/day) adjusted for a mean weight increase of 12 kg during pregnancy. During lactation, approximately 120 mg choline is secreted per day in human milk during the first 6 months of exclusive breastfeeding. Thus, the AI for choline lactating females was set to 520 mg/d (3). The AIs were not further linked to health outcomes in pregnant females or their children.

Choline administered to the mother reaches the mother’s circulation as free choline, betaine, or other derivatives and appears to pass to the fetus or the child via active transport. This is evident from studies showing higher levels of choline and related derivatives in amniotic fluid, cord blood (36, 42, 43), and breast milk compared to mother plasma (44). Using [^3^H]-choline in the dually perfused human placenta has shown that choline perfusion was associated with 4% preferential transport toward the fetal circulation (16).

Maternal plasma concentrations of choline and betaine are subject to dynamic changes during pregnancy (45–47). Concentrations of choline increase in plasma of the females throughout gestation (+50% between first and third trimesters of pregnancy), while betaine levels decline within the same period (by approximately 36%) (45, 47).

Adequate choline intake during early life has been related to growth and normal development of the fetus and child.

The EFSA Panel evaluated two case–control studies for the association between maternal choline intake and neural tube defects (NTDs) in the offspring (48, 49). The association between choline intake and risk of NTDs was inconsistent, and it was recognized that the association may be influenced by the intake of other nutrients and the PEMT genotype of the mother. The data on choline intake and risk of NTDs were not used to derive DRVs for choline. Since the EFSA systematic review of the literature, three additional case–control studies on maternal choline intake/or status and NTDs became available and were entered in a recent meta-analysis. A recent systematic review and meta-analysis of five case–control studies (28, 48–53), showed that low maternal intake or status of choline is associated with higher odds ratio (OR) for NTD [pooled estimate (95% confidence intervals) = 1.36 (1.11, 1.67)]. The 95% prediction intervals were (0.78, 2.36) (54). Some of the studies originated from the US and Canada and were conducted after the fortification with folic acid that could show interaction with choline on the development of the neural tube. Randomized controlled trials (RCTs) using choline (without folic acid) to prevent NTDs are not ethical. The results of the meta-analysis combined with the experimental evidence in animals strongly suggest that the relationship between insufficient maternal choline and the risk of NTD is likely to reflect a causal relationship. Future studies are warranted.

The amount of total choline in breast milk is higher than in maternal blood (55), and it raises by 114% from the stage of colostrum (2–6 days) to 6–7 days postpartum (31). Ilcol et al. found that free choline in breast milk was positively correlated to free choline and choline-containing phospholipids in maternal serum (30). In addition, higher choline and choline-containing derivatives in breast milk were associated with higher levels of serum free choline in the infant (30). Experimental studies in mice suggest that the main part of maternal choline intake is extracted into breast milk (56). This evidence is supported by human studies, showing that higher maternal choline intake from the diet [750 mg choline/d on top of the diet versus placebo] was associated with higher breast milk phosphatidylcholine (44). Therefore, increasing choline intake of lactating women can influence not only maternal serum/plasma choline but also breast milk choline derivatives (44) and thereby choline intake available for the infants and choline status of the infants.

RCTs support positive effects of maternal choline supply on some domains of child neurodevelopment (self-regulation) and neurocognition (learning and memory) (57–59). However, available RCTs have provided higher daily intake of choline than the AI and have measured heterogeneous outcomes. Studies with larger sample size and well-planned outcomes are still warranted.

Non-interventional studies investigated the association between child serum total choline and free choline or choline intake and neurodevelopment/neurocognition at different ages (between 6 months and 7 years) and showed mixed results (34, 60–66), which could be due to measuring non-fasting choline, and the fact that choline levels in blood are not a good estimate of choline status or intake.

### Cognitive function in elderly people

The systematic review conducted by the EFSA panel identified one prospective cohort study that investigated the association between habitual intake of choline and cognitive function in 1,391 men and women (aged 36–83 years) free of dementia at baseline (3). Performances of verbal and visual memory were significantly better with higher choline intake, but there were no significant effects for verbal learning and executive function (67). A recent prospective cohort study with participants from the Kuopio Ischaemic Heart Disease Risk Factor Study, including 2,497 Finnish men aged 42–60 years, examined the relationship between total choline and phosphatidylcholine intake on incidence of dementia (68). After 21.9 years follow-up, higher phosphatidylcholine intake, but not total choline intake, was associated with a decreased risk of dementia (68). Thus, a few observations in healthy adults imply a positive role of dietary choline in cognitive functions, but the prospective data on relationships between choline intake and cognitive function are limited, and no results from intervention studies are available.

### Fatty liver

The effects of choline on the liver have been shown in depletion and repletion studies and in studies among patients receiving parenteral nutrition. Moreover, feeding healthy adult males a choline deficient diet (13 mg/d) for 3 weeks caused 30% lowering of plasma choline and phosphatidylcholine, depletion of choline stores in the liver and elevated serum alanine aminotransferase activity (ALT), suggesting incipient liver damage (4). This effect was averted when the participants received 500 mg/d choline (i.e. plasma choline increased and serum ALT declined) (4). Moreover, patients receiving parenteral nutrition that was depleted of choline had low plasma choline and developed liver steatosis as shown by elevated plasma liver enzyme activities (ALT) (69). Plasma-free choline was increased, and steatosis of the liver declined within 1 to 6 weeks after starting choline supplementation versus the placebo (70, 71). The liver-damaging effect of choline deficiency is likely to be unique and not fully prevented by other methyl donors such as methionine (72). Fatty liver is prevalent in the general population, especially in individuals with overweight or obesity and in patients with type 2 diabetes mellitus. The contribution of insufficient choline intake and status to fatty liver on a population level is not well studied. More studies are warranted because foods rich in choline are also rich in fats, thus making it more challenging to detangle the effect of choline from that of fats and excess nutrition.

### Cardiovascular disease incidence and mortality

The SR by EFSA identified two prospective cohort studies on dietary choline and cardiovascular disease (CVD) incidence (3). These two large prospective cohort studies in men and women without prior CVD did not show a significant relationship between choline intake and risk of CVD (73, 74). EFSA concluded that data on choline intake and risk of CVD cannot be used to derive DRVs for choline (3). This is supported by results from recent prospective cohort studies, showing no associations between choline intake and risk of total CVD, coronary heart disease, stroke, or atrial fibrillation (75–77).

Observations from a few recent cohort studies show conflicting results on dietary choline intake and CVD mortality. In a Japanese cohort of 29,279 men and women, higher intake of total choline and sphingomyelin was associated with no or higher risk of CVD mortality, respectively (78). In two large U.S. cohorts, a higher intake of choline and phosphatidylcholine was associated with increased risk of CVD mortality (12, 79). In a study by Yang et al. (80) including three cohorts from U.S. and China, no association between dietary choline and risk of stroke mortality was reported. An increased risk of ischemic heart disease mortality in the highest quintile of choline intake compared with the lowest was reported for two of the three cohorts with no association in one cohort (80). Thus, a few observations in healthy adults imply a possible positive association of dietary choline with CVD mortality, but results are conflicting. Prospective data on relationships between choline intake and CVD mortality in European populations are lacking, and no results from intervention studies are available.

### Type 2 diabetes mellitus

In a finish cohort of 2,332 men aged 42–60 years from the Kuopio Ischaemic Heart Disease Risk Factor Study, higher baseline total choline and phosphatidylcholine intakes were associated with a lower risk of type 2 diabetes after a mean of 19.3-year follow-up (81). The associations between higher total choline intake and lower risk of diabetes were generally weakened after multiple adjustments, while for phosphatidylcholine, there seems to be a dose-response inverse association, suggesting that the source or the form of choline in the diet could have differential effect on the risk of diabetes. On the other hand, higher intake of phosphatidylcholine was associated with higher risk of type 2 diabetes mellitus in a large U.S. cohort (82). Future RCTs and prospective observational studies may provide better evidence on the association between choline (or phosphatidylcholine) intake and the risk of type 2 diabetes.

### Safety

Side effects reported after using high doses of oral choline [between 7.5 and 20 g/day] were hypotension, gastrointestinal symptoms, and fishy body odor. The lowest choline intake where side effects were observed was 7.5 g/d. Thus, the upper tolerable level of choline was set to 3.5 g/day for adults after the application of an uncertainty factor of 2 (2, 3). Elevated plasma concentration of TMAO has been shown to be associated with renal dysfunction and prevalent CVDs (83, 84). Studies on choline consumptions (i.e. from eggs) (32, 85–87) as determinant of plasma or urinary TMAO show considerable between-individual heterogeneity and generally low (86) or even no effect on TMAO (32, 85, 87), TMAO concentrations show dependency on gut bacteria dependency on gut microbiota (88, 89), choline source (supplements versus diet) (90), and choline form (88). Choline intake from eggs, for instance, failed to show an effect on gut microbiota (87) and also no firm evidence on cardiovascular risk. It remains unclear whether TMAO is a result or a culprit of CVD or related clinical conditions such as renal dysfunction.

At present, we consider TMAO as inappropriate outcome to set the UL for choline intake. We recommend considering the UL of choline of 3.5 g as defined by the EFSA and IOM. For instance, in order to exceed the 3.5 g/day intake of choline, a person must eat 2.94 kg Salmon per day. Exceeding this intake through a natural diet on long term is very unlikely, and most available supplements provide between 100 mg and 1 g choline on top of the natural diet.

## Requirement and recommended intakes

It is generally well recognized that the present AIs defined by IOM and EFSA of choline are not achieved through the diet on a population level. It is also recognized that the AIs were not related to clinical health outcomes. The lack of food composition databases, data on food dietary intakes, and optimal biomarkers make the interpretation and generalizability of results from observational studies difficult. Interventional studies with controlled choline intake or RCTs (e.g. with appropriate comparator) are the most reliable way to link choline to health outcomes. Also, studies showing a dose-response relationship are needed to strengthen the present evidence on the role of choline in some outcomes, such as maternal-child health or cognitive function in elderly people. Most of the available RCTs on maternal supplementation and child neurocognition or neurodevelopment have limitations. However, most RCTs achieved total choline intakes (diet plus supplements) of 1 g/d or higher, suggesting that a possible positive effect of choline on brain function may be expected at levels that are almost twice as high as the present AI for pregnant and lactating women.

There are several gaps in knowledge in the field of choline. For instance, there are polymorphisms in enzymes involved in choline and folate metabolisms that could interfere with choline requirements. In addition, because animal foods are the main source of choline in the diet, some of the associations between choline intake and health outcomes could be abolished due to other components in the same food sources such as fats. Moreover, the health effects of choline need further investigations since they might differ depending on the choline forms that, in turn, could influence bioavailability and metabolic path. There could be interactions between choline, folate, and vitamin B_12_, and thus, choline could in theory show stronger effects on health outcomes in people with low folate status, while its role becomes less important when folate status is high.

### Reasoning behind the recommendations

Choline intake recommendations by the EFSA and IOM were based on depletion-repletion studies among adults who showed liver damage after cutting choline from the diet, and this sign was corrected after administering 500 mg choline/d. Intervention studies in pregnant females using 480 mg/d (vs. 960 mg/d) showed no consistent effect on health outcomes, thus supporting the view that 480 mg/d was sufficient to maintain health. According to the EFSA and IOM, the AI of choline for pregnant females (480 and 450 mg/d) and lactating females (520 and 550 mg/d) is similar. The recommendations of sufficient choline intake for pregnant and lactating females as suggested by the EFSA appear to be justified. However, intakes above this level maybe needed for brain development, implying that pregnant and lactating females may need to achieve higher choline intake through supplements.
